# Histomorphometric, Immunohistochemical and Microtomographic Comparison between Autogenous and Xenogenous Bone Blocks for Mandibular Lateral Augmentation in Rabbits

**DOI:** 10.3390/ma14206049

**Published:** 2021-10-13

**Authors:** Erick Ricardo Silva, Vitor Ferreira Balan, Daniele Botticelli, Claudio Soldini, Roberta Okamoto, Samuel Porfirio Xavier

**Affiliations:** 1 Department of Oral and Maxillofacial Surgery and Periodontology, Faculty of Dentistry of Ribeirão Preto, University of São Paulo, Ribeirão Preto 14040-904, Brazil; erickricardo.rp@gmail.com (E.R.S.); vitor.balan@usp.br (V.F.B.); spx@forp.usp.br (S.P.X.); 2 Ariminum Research & Dental Education Center, 47923 Rimini, Italy; 3 Institute for Dental & Implant Studies (IDIS), 36100 Vicenza, Italy; claudiosoldini.it@gmail.com; 4 Department of Basic Science, Division of Anatomy, Araçatuba Dental School, Universidade Estadual Paulista “Júlio de Mesquita Filho” (UNESP), Araçatuba 16015-050, Brazil; roberta.okamoto@unesp.br

**Keywords:** histomorphometry, microtomography, immunohistochemistry, xenograft, rabbit

## Abstract

Background: The volumetric and biological behaviors of equine block grafts compared with autogenous block grafts have not yet been assessed. Hence, the aim of the present study was to compare—by means of histomorphometry, immunohistochemistry and microtomography—the graft incorporation and remodeling processes of autogenous and equine xenogenous bone blocks used for mandibular lateral augmentation in rabbits. Methods: Autogenous bone grafts harvested from the iliac bony crest and equine block grafts were secured to the lateral aspect of the mandible angle of eighteen rabbits. The healing after 7, 20 and 60 days was assessed in six animals each period. Results: After 60 days, new bone was present 24.2 ± 11.2% and 31.6 ± 13.3% in the autograft and xenograft groups, respectively. A better integration to the recipient sites was observed in the autogenous compared with the xenogenous blocks. Conclusions: Both xenogenous and autogenous bone blocks presented similar percentages of newly formed bone over time. However, bone volume, the quality of the grafted area and graft incorporation to the recipient sites were superior in the autogenous compared with the equine xenogenous graft sites.

## 1. Introduction

A sufficient volume of alveolar bone is required for an implant placement to achieve a predictable prognosis over time [1]. However, when necessary, the bone volume might be increased using several different techniques [1,2,3,4,5,6].

A recent systematic review with a meta-analysis of lateral bone augmentation showed that autogenous bone blocks are the most frequently used technique for bone reconstruction [7]. For this technique, a mean horizontal bone gain of 4.25 mm was observed. Another systematic review with a meta-analysis reported a horizontal bone gain of 3.7 mm from the grafting procedure and a width loss of less than 1.0 mm six months later, as evaluated during the implant placement. Implant success rates of 98.9% to 100% were obtained using this technique [8].

A lateral bone augmentation with autologous bone grafts yields optimal outcomes [7,8]. However, its usage has been associated with several disadvantages mainly related to the addition of a donor site [6,9,10,11,12]. A volumetric reduction of the autogenous grafts over time has been reported, especially in the first five years after the implant insertion [6]. Hence, the use of blocks of allografts, xenografts and alloplastic grafts has been suggested as an alternative for mandibular lateral augmentation. 

Corticocancellous fresh-frozen allografts used for augmenting atrophic posterior mandibles in humans has presented approximately 20% of new bone formation after six months [13]. Nevertheless, a volumetric contraction of about 40% was observed after the first year of implant functional loading [14]. 

Xenogenous bone has been proposed as a reliable procedure for a lateral bone augmentation. A systematic review of different techniques using xenografts has shown a mean horizontal bone gain of 4.4 mm [15]. Most of the studies included particulate deproteinized bovine bone mineral as the xenograft. In a randomized, controlled, split-mouth, prospective clinical trial comparing autografts and xenografts in blocks for an anterior maxillary horizontal augmentation, it was demonstrated that xenografts present a suitable volumetric stability over time, allowing the installation of implants with reduced insertion torques [16]. However, the data on xenografts are controversial with several studies showing excellent results [17,18] and others with an insignificant amount of newly formed bone [19,20,21]. 

Equine xenografts have been recently introduced to reconstructive procedures. A randomized, parallel, double-blind clinical trial evaluated new bone formation after a maxillary sinus augmentation with bovine and equine bone [22]. Similar amounts of newly formed bone (~22%) were reported for both groups six months after the surgery. A recent systematic review has shown an estimated mean of newly formed bone of 22.74% for bovine and 44.51% for equine xenografts, respectively [23]. These data suggest that equine bone appears to be as effective as bovine bone for sinus floor augmentations. However, there are few studies on the use of equine bone for mandibular lateral augmentations.

In a clinical series of cases, mandibular ridge defects were augmented using onlay appositional equine bone blocks protected by a membrane reinforced by titanium [24]. After six months, biopsies were taken for histological and immunohistochemical evaluations. A horizontal gain from ≤4.0 mm to ≥7.0 mm and a percentage of 35% of newly formed bone were observed. Computed tomography scans were only used for linear measurements. 

Experimental studies have also assessed the applicability of equine bone for mandibular augmentation in different animal models [25,26,27]. In a study with dogs, bovine and equine blocks were used for reconstructing four standardized box-shaped defects created at the buccal aspect of the mandible of five animals [25]. After 12 months of healing, it was not possible to find differences between the groups for newly formed bone. In another study, rats underwent a lateral mandible augmentation with autogenous bone (iliac crest), bovine bone or equine bone [27]. A higher bone formation was observed for the equine group compared with the bovine group but it was lower than the autogenous group after 1 and 3 months of healing. Although there are comparative studies on grafts for mandibular augmentation, this is the first investigation on equine versus autogenous bone from incorporation to volumetric remodeling, as we have already achieved for auto and allografts.

Thus, the aim of the present study was to compare, by means of histomorphometry, immunohistochemistry and microtomography, the graft incorporation and remodeling processes of autogenous and equine bone blocks used for mandibular lateral augmentations in rabbits.

## 2. Materials and Methods

### 2.1. Animal Sample

Eighteen New Zealand male white rabbits (weight ~3.5–4.0 kg, age 4–5 months) were divided into three groups of six animals and assigned to 7, 20 and 60 days of evaluation, respectively.

The sample size of 6 was determined based on the data from similar studies that disclosed statistically significant differences in new bone percentages from that number of rabbits.

### 2.2. Study Design and Randomization

The angle of the mandible was bilaterally used as the recipient site. Either autogenous bone grafts harvested from the anterior iliac bony crest or a xenograft were randomly fixed on the lateral aspect of the mandible. The randomization was carried out by an author (D.B.) using a website [28]. The surgeon received the information of the allocation of the graft type after the iliac bone graft had been removed and the first recipient sites prepared.

The different conformation of the block did not allow for a blinding for the histological assessment.

### 2.3. Surgical Procedure

The anesthesia included 1.0 mg/kg of acepromazine SQ (Acepran^®^, Vetnil, Louveira, São Paulo, Brazil), 3.0 mg/kg of xylazine IM (Dopaser^®^, Hertape Calier, Juatuba, Minas Gerais, Brazil) and 50 mg/kg of ketamine IM (União Química Farmacêutica Nacional S/A, Embuguaçú, São Paulo, Brazil). Moreover, 40 mg/kg of oxytetracycline IM (Biovet, Vargem Grande Paulista, São Paulo, Brazil) was inoculated. Mepivacaine (2%) and epinephrine (1:100.000) (Mepiadre, Nova DFL, Rio de Janeiro, Brazil) were injected locally.

**Figure 1:**
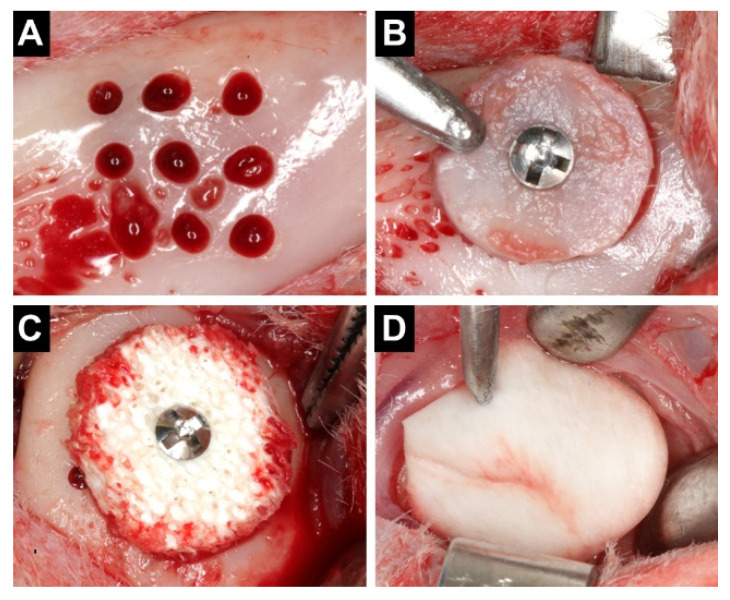
Surgical procedures. (**A**) Nine equidistant monocortical perforations at the recipient site. (**B**) Autogenous bone block fixed to the angle of the mandible. (**C**) Equine xenogenous bone block fixed at the contralateral recipient site. (**D**) Bovine pericardium membrane to cover the grafts.

After an incision of the soft tissues on the protuberance of the anterior iliac bony crest, a trephine (Neodent, Curitiba, Paraná, Brazil) with 10 mm of internal diameter was used to harvest a bicortical bone block. The bone graft was maintained in a gauze soaked in sterile saline and the wounds were closed with internal resorbable stitches and nylon external sutures. An incision was subsequently performed on the lateral aspect of the mandible angle and nine perforations of a 1.0 mm diameter were created using a template (Figure 1A). The inner cortical layer of the bone block was removed and the graft was remodeled to obtain a good passive adaptation to the recipient site. A titanium screw (Neodent, Curitiba, Paraná, Brazil), 8 mm long and 1.5 mm in diameter, was used to secure the block to the recipient site (Figure 1B). At the opposite side, an equine spongious bone block (Heket plate, Heket Biomaterials, Trento, Italy) composed of 30% hydroxyapatite and 70% bone collagen partially demineralized in an acid solution and enzymatically deantigenated at 37 °C was used. The xenogeneic block was prepared with identical dimensions to the autogenous bone block and fixed with a titanium screw (Figure 1C). Both grafts were protected with bilayer non-cross-linked bovine pericardium membranes (Exaflex matrix, Heket Biomaterials, Trento, Italy, Figure 1D) and the wound was closed with sutures. Ketoprofen (3.0 mg/kg IM) (Ketofen^®^ 10%, Merial, Campinas, São Paulo, Brazil) and tramadol (2% 1.0 mg/kg SQ) (Cronidor, Agener União Saúde Animal, Apucarana, Paraná, Brazil) were administered twice a day for two days during the post-operative period.

### 2.4. Maintenance Care

Individual cages in acclimatized rooms were used to host the animals during the whole period of the experiment. The biological functions and the wounds were inspected daily by professionals.

### 2.5. Euthanasia

The animals were euthanized with an intravenous overdose (2.0 mL) of thiopental 1.0 g (Thiopentax^®^, Cristália, Itapira, São Paulo, Brazil).

### 2.6. Micro-CT Evaluations

The biopsies containing the blocks were fixed in formalin and then scanned using a high-resolution micro-CT SkyScan 1172 (Bruker, Kontich, Belgium). The following parameters were used: a resolution of 10.97 µm, isotropic pixels at 100 kV/100 µA with an Al + Cu filter, exposure 1280 ms, rotation step 0.6°, a frame average of 4 and random movement of 10.

**Figure 2:**
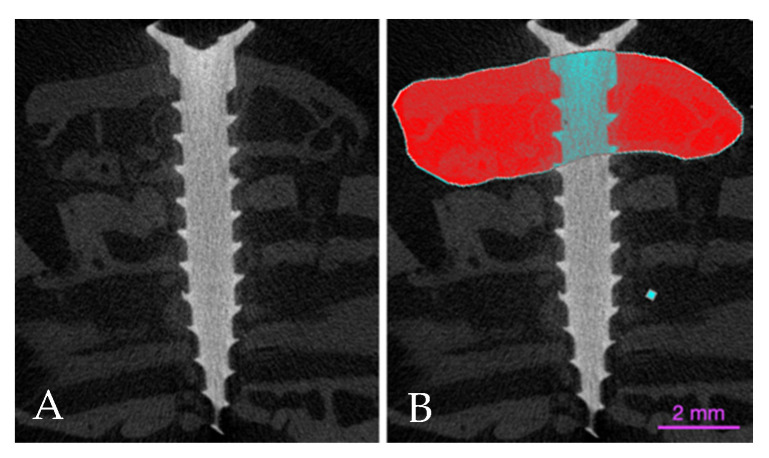
(**A**) Augmented site. (**B**) Volume analyzed (red).

A DataViewer (Bruker, Kontich, Belgium, Figure 2) was used to reposition the cross-sectional images and CTAn (Bruker, Kontich, Belgium) was applied to evaluate the volume of the grafted regions. The fixation screw was eliminated from the measurements. The thresholds of gray levels to identify the total graft volume (TV) and bone volume (BV = new bone plus residual graft) were set at 30–180 gray levels.

### 2.7. Histological Preparation

Following the micro-CT analyses, the specimens were decalcified with 4% EDTA (Merck, Darmstadt, Germany) and subsequently dehydrated through a series of alcohols with increasing concentrations. Following this, the specimens were diaphanized with xylol immersions and impregnated with paraffin (Leica TP 1020, Wetzlar, Germany). The paraffin blocks containing the samples were cut in a microtome to obtain slides 6 µm thick. The slides were divided in two groups for either histological or immunohistochemical analyses. The slides for the histological analysis were stained with hematoxylin and eosin or Masson’s Trichrome stain.

### 2.8. Calibration of the Histomorphometric Evaluation

A trained examiner (E.R.S.) performed the analysis after a calibration with an experienced professional (D.B.). The inter-examiner test for the recognition of histological structures reached a K > 0.90.

### 2.9. Histological and Histomorphometric Analyses

All histological evaluations were performed using a light microscope (Leica Microsystems, Bensheim, Germany) connected to a computer through a digital video camera (Leica DC 300F, Leica Microsystems, Bensheim, Germany). Image J 1.50i software (National Institutes of Health, Bethesda, MD, USA) was used for the measurements. A point-counting procedure was applied to determine the tissue composition and grids consisting of 80 squares were superposed onto the image of the histological slides.

**Figure 3:**
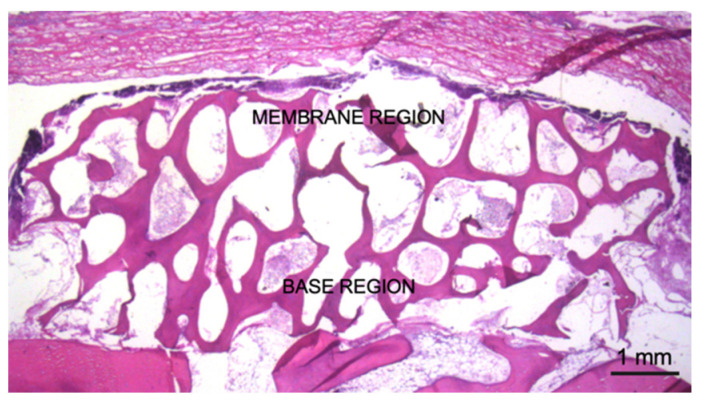
Membrane and Base regions analyzed histologically.

The area delineated by the upper portion of the graft in contact with the collagen membrane was defined as the Membrane region and the area close to the recipient bed was defined as the Base region (Figure 3). The evaluations were performed using an objective 20×.

The histomorphometric measurements were taken separately for each area and the mean values were calculated to evaluate the new bone formation and graft resorption. The structures assessed included proportions of the new mineralized bone and remaining graft (composed of pre-existing mineralized bone and marrow spaces).

### 2.10. Immunohistochemical Processing

The immunohistochemical labeling was carried out using the immunoperoxidase detection method with the following primary antibodies: anti-Col I (COL1), anti-OPN (AKM2A1), anti-VEGF (SC1881), anti-ALP (B-10) and anti-Cas 3 (31A1067) polyclonal antibodies produced in goats (Santa Cruz Biotechnology, Santa Cruz, CA, USA). As a secondary antibody, the anti-IgG biotinylated antibody at a 1:200 concentration (Jackson Immunoresearch Laboratories, West Grove, PA, USA) was used. The reaction was revealed using diaminobenzidine (Dako Laboratories, Santa Clara, CA, USA). At the end of the reactions, Harris hematoxylin counter-staining was performed. The determination of the labeling levels for each antibody was performed semi-quantitatively using scores from 0 to 3 (0 = absence of labeling, 1 = light labeling in which up to 25% of the analyzed area showed positive labeling for the protein investigated, 2 = moderate labeling in which 50% of the analyzed area presented positive labeling for the protein, 3 = intense labeling in which up to 75% of the analyzed area showed positive labeling for the analyzed protein).

The analysis was performed using a light microscope (Leica Microsystems, Bensheim, Germany) connected to a computer through a digital video camera (Leica DC 300F, Leica Microsystems, Bensheim, Germany) using objectives 10× and 25×. The analyzer (RO) had no previous knowledge of the samples and was submitted to the Kappa intra-examiner test (K > 0.80).

### 2.11. Statistical Analysis

IBM SPSS statistical software (IBM Inc., Chicago, IL, USA) was used for statistical analyses and the mean values and standard deviations were reported. The normal distribution was assessed with the Shapiro–Wilk test. The new bone formation was the primary variable. A two-way ANOVA test was used to evaluate the outcomes among the periods. Differences between the groups were assessed using a Student’s *t*-test. The level of significance was set at 5% (*p* < 0.05). In the case of a statistically significant difference, a Tukey post-test was applied.

## 3. Results

### 3.1. Animal Conditions

One rabbit died during anesthesia and it was replaced. No other animals experienced a complication so an *n* = 6 was attained for all periods of analyses.

### 3.2. Micro-CT Evaluation

**Figure 4:**
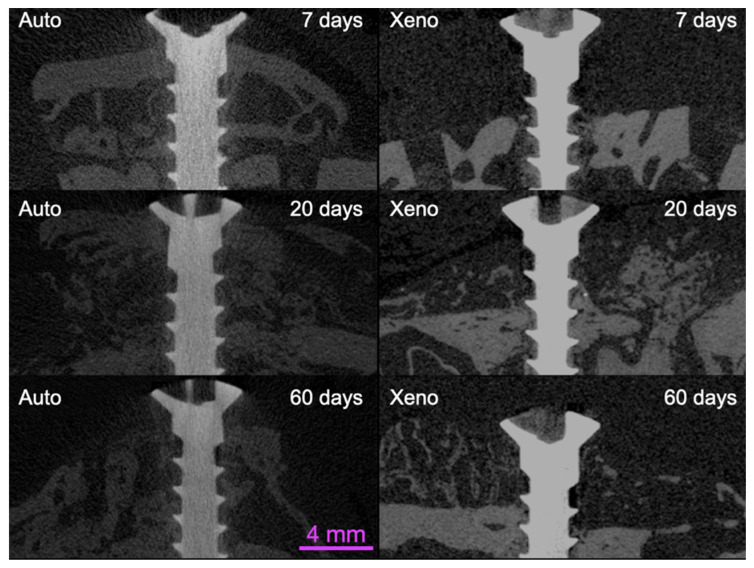
Cross-sectional microtomographic images of the autogenous (control) and xenogenous (test) groups showing the volumetric changes of the grafts after 7, 20 and 60 days of healing.

**Figure 5:**
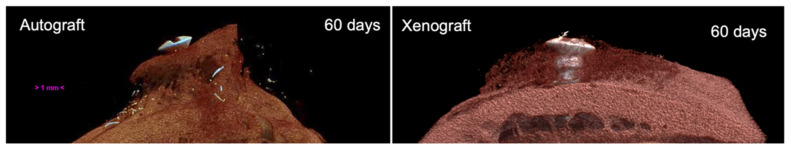
Micro-CT images representing the healing after 60 days in the autogenous (control) and xenogenous (test) groups.

For more detailed information about the graft and bone volume changes over time, bone to implant contact to the titanium screw and linear bone gain, see another report on the same material [29]. The total volume (TV) reduced between 7 and 60 days from 258.13 ± 15.3 mm^3^ to 107.2 ± 17.5 mm^3^ (*p* = 0.001) for the autogenous bone grafts and 166.9 ± 26.0 mm^3^ to 96.0 ± 16.1 mm^3^ (*p* = 0.022) for the xenografts (Figure 4 and Figure 5; Table 1). 

The autogenous bone grafts presented a bone volume (BV: new bone plus residual graft) of 52.8 ± 7.7 mm^3^, 27.2 ± 11.1 mm^3^ and 33.1 ± 2.8 mm^3^ after 7, 20 and 60 days of healing, respectively. The xenograft bone volumes were 4.2 ± 0.4 mm^3^, 18.8 ± 9.5 mm^3^ and 7.6 ± 3.7 mm^3^, respectively. The inter-group analysis disclosed a higher bone volume in the autogenous bone grafts compared with the xenografts after 60 days of healing (*p* = 0.003).

### 3.3. Histomorphometric Evaluation

**Figure 6:**
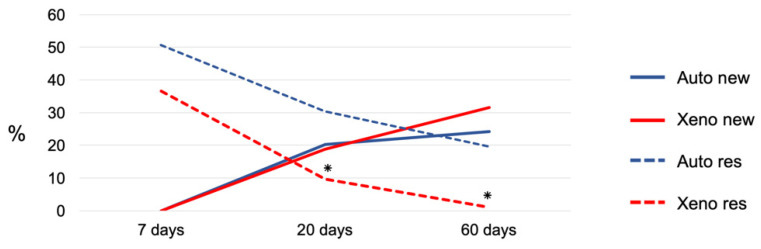
Graph illustrating the percentages of new bone and residual grafts in the xenogenous (test) and autogenous (control) groups after 7, 20 and 60 days of healing. * *p* < 0.05 inter-group analysis.

After 7 days of healing, new bone was not observed in either group (Figure 6; Table 2). 

**Figure 7:**
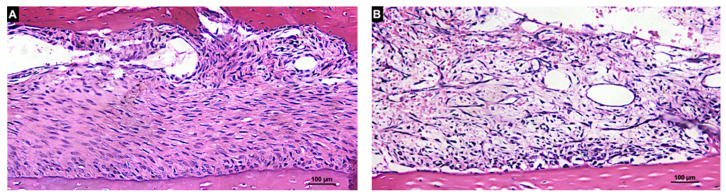
Histologic photomicrography of the interface between the recipient bed and graft (Base region) in the autogenous (**A**) and xenogenous (**B**) grafts after 7 days of healing. A provisional matrix rich in fibroblast-like cells was observed for both groups. Hematoxylin and eosin stain; magnification 200×.

In the Base region in both groups, a provisional matrix rich in fibroblast-like cells, vessels and fibers was found interposed between the graft and the cortical bone of the recipient sites (Figure 7A,B). In the Membrane region, the collagen membrane lined the grafts in both the autogenous and xenogenous grafts and no new bone was found.

**Figure 8:**
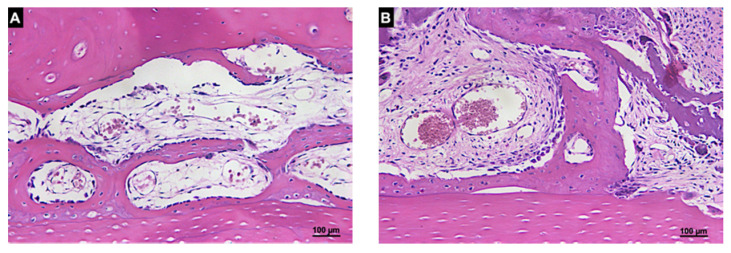
Histologic photomicrography of the Base region in the autogenous (**A**) and xenogenous (**B**) graft after 20 days of healing. New bone was found forming bridges between the grafts and the cortical bone of the recipient site in both groups. Hematoxylin and eosin stain; magnification 200×.

After 20 days of healing, 20.3 ± 13.8% and 18.9 ± 4.4% of new bone were found in the autogenous and xenogenous graft groups, respectively (Table 2, Figure 6). In the Base regions of the autogenous bone, the new bone formed bridges connecting the graft to the recipient cortical bone (Figure 8A) as well as in the Base region of the xenogenous bone (Figure 8B). 

**Figure 9:**
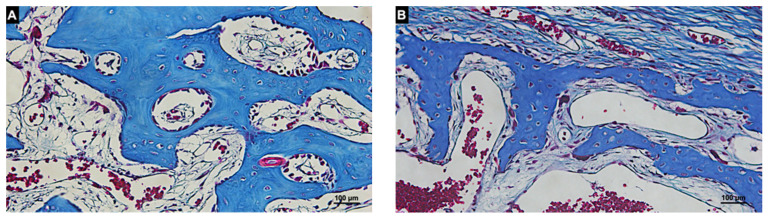
Histologic photomicrography of the Membrane region in the autogenous (**A**) and xenogenous (**B**) graft after 20 days of healing. New bone was found forming from the graft surface in the autogenous group and in close contact to the trabeculae of the graft in the xenogenous group. Masson’s Trichrome stain; magnification 200×.

In the Membrane regions, new bone was found in both groups. In the autogenous group, the bone formed from the graft surface (Figure 9A) whereas in the xenograft group, the new bone was found within the trabeculae of the xenograft (Figure 9B).

**Figure 10:**
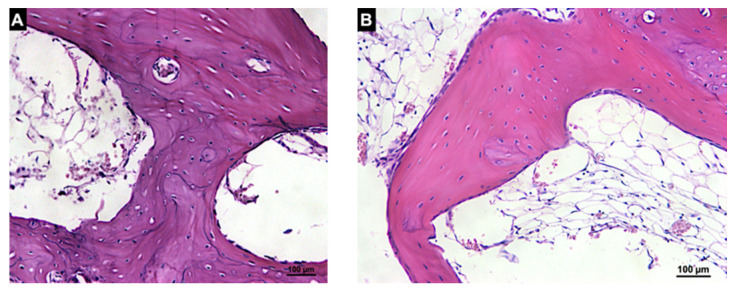
Histologic photomicrography of the Base region in the autogenous (**A**) and xenogenous (**B**) grafts after 60 days of healing. The autografts were well-incorporated into the recipient bed and the xenografts were connected to the cortical bone through sparse bone bridges. Hematoxylin and eosin stain; magnification 200×.

After 60 days, the new bone increased in both groups reaching fractions of 24.2 ± 11.2% and 31.6 ± 13.3% in the autograft and xenograft groups, respectively (Table 2, Figure 6). The bone graft was remodeled in most sites and regions with empty lacunae were seen in the residual bone graft. The xenograft appeared to be mainly resorbed and substituted by the new bone and marrow spaces. Nevertheless, regions presenting immature tissues (provisional matrix) were still observed. In the Base region of the autogenous bone, the grafts were connected and well-integrated to the recipient sites (Figure 10A). The xenograft was connected by means of sparse bridges of newly formed bone to the recipient sites (Figure 10B).

**Figure 11:**
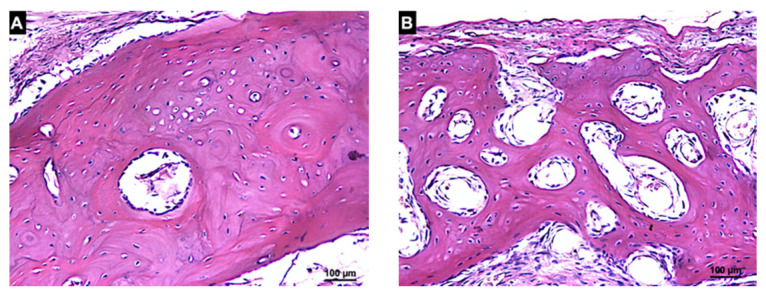
Histologic photomicrography of the Base region in the autogenous (**A**) and xenogenous (**B**) grafts after 60 days of healing. The autogenous bone was remodeled and presented secondary osteons with rare empty lacunae. The new bone in the xenografts was mainly represented by primary osteons. Small amounts of the remnants of the xenograft were detected. Hematoxylin and eosin stain; magnification 200×.

In the Membrane region, the autogenous bone was remodeled and presented secondary osteons with occasional empty lacunae (Figure 11A) whereas in the xenograft group (Figure 11B), the new bone contained primary osteons and small amounts of the remnants of the xenograft could be detected.

The collagen (bovine pericardium) membranes used to cover the grafts were gradually resorbed. At 60 days, the remnants of the membrane were hardly visible.

### 3.4. Immunohistochemical Evaluation

**Figure 12:**
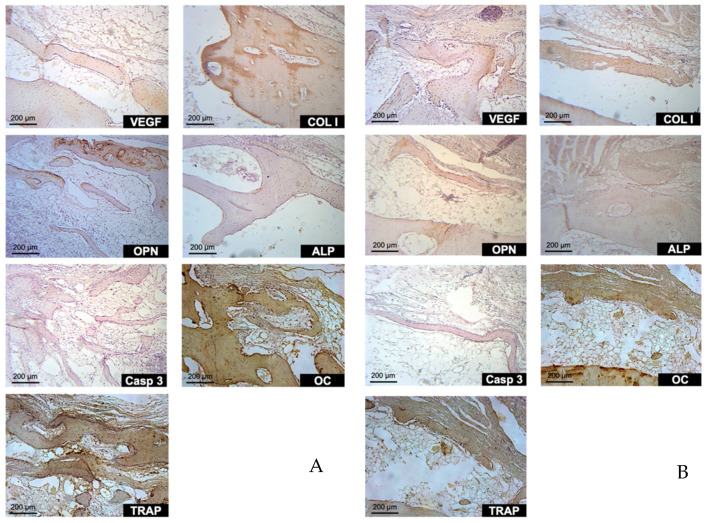
Autogenous (**A**) and xenogenous (**B**) groups after 60 days. Immunolabeled proteins used in this study: VEGF: vascular endothelial growth factor; COL I: collagen type I; OPN: osteopontin; ALP: alkaline phosphatase; Casp 3: caspase 3; OC: osteocalcin; TRAP: tartrate-resistant acid phosphatase. Magnification 200×.

VEGF labeling showed higher scores for the control group (autogenous; Figure 12A) compared with the test group (Figure 12B) mainly in the initial periods of incorporation of the grafts; i.e., 7 and 20 days. Collagen Type I presented a similar pattern of labeling between the test and control groups with a slightly higher score for the autogenous bone, especially at 7 and 60 days (Table 3). 

For osteopontin, it was not possible to observe any differences between the test and control groups for all the evaluation periods (Figure 12A,B; Table 3). ALP presented slightly higher scores for the test compared with the control group, especially in the last evaluation period. The same was observed for Caspase 3 in which the test group also presented higher scores for the initial evaluation period; i.e., 7 days. 

TRAP, a protein strictly related to bone resorption, presented higher scores for the autogenous bone compared with the xenogenous bone, especially after 60 days of healing. A similar immunolabelling pattern was also observed for OC.

## 4. Discussion

The autogenous bone grafts were completely integrated into the recipient sites after 60 days of healing. An approximate 53% loss of volume was observed compared with the 7-day period. The xenograft was almost completely resorbed and only partly substituted by the new mineralized bone, mainly at the base and in the periphery.

The autogenous bone grafts were resorbed at the periphery while, within the body, remodeling processes were occurring. New bone formation was seen incorporating the graft over time in the interface between the graft and the recipient site.

The results obtained from the autogenous bone sites were in agreement with other studies that used autogenous bone for onlay bone augmentation. In a similar experiment in rabbits [30], autogenous bone grafts harvested from the iliac crest were placed on either side of the mandible. However, only one recipient site received a perforation of the cortical layer and the contralateral side was left untreated. The incorporation of the graft at the perforated sites was optimal and 50% of the volume was lost. At the untreated site, the graft presented a 70% volume loss and a poor incorporation into the recipient site. In another similar experiment in rabbits [31], bicortical autogenous bone was harvested from the calvaria and applied to the lateral aspect of the mandible, either perforated or not. After 60 days, the resorption was higher at the not-perforated sites (~22%) compared with the perforated sites (~6%) even though both grafts were incorporated into the recipient bed.

The recipient bed preparation by means of perforations has been shown to accelerate and improve the incorporation process. In the studies previously mentioned [30,31], a 20-day interval was evaluated and faster bone formation and graft incorporation were observed at the perforated sites. After 3 days of healing, VEGF was only present at the perforated sites. In the present study, after 7 days of healing, a tissue including fibroblast-like cells, vessels and fibers was observed within the perforations and the first bone formation was seen after 20 days. These results agreed with another similar study [32] in which autografts from the calvaria were fixed to the lateral aspect of the mandible. The healing at the interface region between the autograft and the recipient site was assessed after 3, 7, 20 and 40 days. Similar to the present study, after 7 days of healing, a tissue rich in fibroblast-like cells, vessels and fibers was observed within the perforations at the recipient bed and new bone was detected after 20 days.

In the present study, a high rate of remodeling of the autograft was observed. The remodeling process of autogenous grafts has been reported in previous studies [29,30]. In an experiment in dogs [20], an autogenous bone graft was collected from the ascending ramus of the mandible and fixed within the chronic buccal defects of the mandible. After 6 months of healing, the grafts were histologically analyzed. Very little content of no-vital bone was found, indicating that a high rate of remodeling occurred during healing that substituted the pre-existing bone with newly formed bone. In the present study, after 60 days of healing, only ~20% of the pre-existing bone was still present whereas ~24% of the new bone was occupying the augmented region. This, in turn, indicated that additional time was needed to complete the remodeling process.

The micro-CT results showed a better volumetric maintenance over time for the autogenous blocks compared with the xenogenous group. This result might be related to the higher content of residual grafts in the autogenous compared with the xenogenous groups. It has to be considered that the xenograft used in the present study was composed of a poor mineralized structure, which may have contributed to the contraction of the grafts. In the xenograft group, new bone was mainly found in the base region and in the periphery of the membrane region; the most central regions were occupied by soft tissue resembling marrow.

The microtomographic results were expressed only as total bone volume (new bone + remaining graft). The reason has to be related to the difficulties to find the most suitable threshold of greys that might allow for an accurate distinction between the new bone and the graft residuals. The difficulties found here were consistent with those discussed in another study on a micro-CT evaluation of a xenogenous particulate graft [33,34].

Both graft materials used in the present study were extensively resorbed. Other biomaterials have shown a high resorption rate. In a previous prospective study [14], a fresh-frozen allograft was used to augment the lateral aspect of the mandible in twenty patients. CBCTs were taken before surgery and after 1 week, 6 months and 18 months. The volumetric loss of volume was 41%. Nevertheless, another biomaterial presented a much lesser rate of resorption. In the experiment on dogs already mentioned, at one side of the mandible a DBBM block was used to augment a buccal bone defect [20]. After 3 months of healing, the dimensional measurements did not reveal changes compared with the initial surgical stage. In the present study, the incorporation of the xenograft was partial and was composed of a few bridges of newly formed bone connecting the graft to the recipient sites. This feature of healing at the xenograft sites was completely different from that observed at the autogenous sites that presented a more compact connection between the grafts and recipient beds. In the previously described studies [20,21], the graft was completely incorporated into the recipient site in the autogenous sites. However, in the DBBM sites, the blocks were separated from the recipient sites by connective tissue presenting no direct contact to the recipient sites. Occasionally, the grafts were incorporated into the recipient bed and only in a limited region of the base of the grafts.

Finally, the amount of newly formed bone after 60 days in the xenogenous group (31.6. ± 13.3) was higher than that reported for both bovine [20,21,35] and equine bone [27] used for mandibular lateral augmentation.

## 5. Conclusions

The findings from the present study suggest that both xenogenous and autogenous bone blocks present similar percentages of newly formed bone over time. However, bone volume, the quality of the grafted area and graft incorporation to the recipient sites were superior in the autogenous compared with the equine xenogenous graft sites.

## Figures and Tables

**Table 1:** Micro-computed tomographic evaluation of the total volume and bone volume in the various periods of healing. TV: total graft volume; BV: bone volume = new bone + residual graft.

		TV	BV
7 days	Auto	258.3 ± 15.8 *	52.8 ± 7.7 *
	Xeno	166.9 ± 26.0 *	4.2 ± 0.4
20 days	Auto	159.5 ± 77.1	27.2 ± 11.1
	Xeno	166.4 ± 50.3	18.8 ± 9.5
60 days	Auto	107.2 ± 17.5 *	33.1 ± 2.8 ***
	Xeno	96.0. ± 19.1 *	7.6 ± 3.7 ***

Percentages expressed as mean ± standard deviation: * *p* < 0.05 intra-group analysis, *** *p* < 0.05 for both the intra- and inter-group analysis.

**Table 2:** Percentages of new bone and residual grafts obtained from the histomorphometric analysis of the evaluated periods.

		NB	RG
7 days	Auto	0	50.7 ± 24.5
	Xeno	0	36.7 ± 7.9
20 days	Auto	20.3 ± 13.8	30.4 ± 15.4 **
	Xeno	18.9 ± 4.4	9.7 ± 9.9 **
60 days	Auto	24.2 ± 11.2	19.7 ± 10.3 **
	Xeno	31.6. ± 13.3	1.0 ± 1.7 **

Data are expressed as mean ± standard deviation: ** *p* < 0.05 inter-group analysis.

**Table 3:** Scores obtained for the immunolabeled proteins used in this study. 0: absence; 1: light; 1.5: light to moderate; 2: moderate; 2.5: moderate to intense; 3: intense.

		VEGF	COL I	OPN	ALP	Casp 3	OC	TRAP
7 days	Auto	2	2	1	2	1	1.5	1.5
	Xeno	1.5	1.5	2	1.5	2	2	1
20 days	Auto	2	1.5	2.5	1.5	1.5	2	2
	Xeno	1	1.5	1.5	2	2	2	2
60 days	Auto	1	2	2	1	1	2.5	2.5
	Xeno	1.5	1.5	1.5	1.5	2	2	1

## Data Availability

The data are available under reasonable request.
